# Bacterial and viral assemblages in ulcerative colitis patients following fecal microbiota and fecal filtrate transfer

**DOI:** 10.1093/ismeco/ycae167

**Published:** 2024-12-23

**Authors:** Howard Junca, Arndt Steube, Simon Mrowietz, Johannes Stallhofer, Marius Vital, Luiz Gustavo dos Anjos Borges, Dietmar H Pieper, Andreas Stallmach

**Affiliations:** Microbial Interactions and Processes Research Group, Helmholtz Centre for Infection Research, D-38124 Braunschweig, Germany; Department of Internal Medicine IV (Gastroenterology, Hepatology and Infectious Diseases), University Hospital Jena, D-07747 Jena, Germany; Department of Internal Medicine IV (Gastroenterology, Hepatology and Infectious Diseases), University Hospital Jena, D-07747 Jena, Germany; Department of Internal Medicine IV (Gastroenterology, Hepatology and Infectious Diseases), University Hospital Jena, D-07747 Jena, Germany; Microbial Interactions and Processes Research Group, Helmholtz Centre for Infection Research, D-38124 Braunschweig, Germany; Microbial Interactions and Processes Research Group, Helmholtz Centre for Infection Research, D-38124 Braunschweig, Germany; Microbial Interactions and Processes Research Group, Helmholtz Centre for Infection Research, D-38124 Braunschweig, Germany; Department of Internal Medicine IV (Gastroenterology, Hepatology and Infectious Diseases), University Hospital Jena, D-07747 Jena, Germany

**Keywords:** microbiota, virome, ulcerative colitis, fecal microbiota transfer, fecal microbiota filtrate transfer

## Abstract

Fecal microbiota filtrate transfer is discussed as a safe alternative to fecal microbiota transfer (FMT) to treat ulcerative colitis. We investigated modulation of viral and bacterial composition during fecal microbiota filtrate transfer followed by FMT in six patients with active ulcerative colitis (where clinical activity improved in three patients after filtrate transfer) and combined 16S ribosomal RNA gene amplicon sequencing with a virome analysis pipeline including fast viral particle enrichment and metagenome mapping to detect frequencies of 45,033 reference bacteriophage genomes. We showed that after antibiotic treatment and during filtrate transfer, the bacterial community typically adopted a stable composition distinct to that before antibiotic treatment, with no change toward a donor community. FMT in contrast typically changed the bacterial community to a community with similarity to donor(s). There were no indications of an establishment of predominant donor viruses during filtrate transfer but a remodeling of the virome. In contrast, the establishment of donor viruses during FMT correlated with the predicted hosts established during such transfer. Our approach warrants further investigation in a randomized trial to evaluate larger therapeutic interventions in a comparable and efficient manner.

## Graphical Abstract

**Graphical Abstract:**
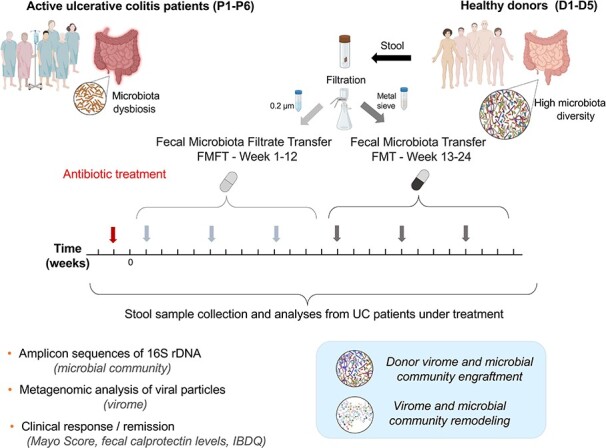

## Introduction

The understanding of ulcerative colitis (UC) has steadily grown in recent decades, and different therapies have been standardized [1]. Clinical observations indicate a close connection between a dysbiotic intestinal microbiota and the initial manifestation and clinical course of UC [2]. Patients have a less diverse microbial community compared to healthy subjects [3] and are thought to be characterized by a decline in short chain fatty acid (SCFA) producing [3] and an increase in pro-inflammatory bacteria [4]. However, the causal relationship between dysbiosis and UC is still unclear. Fecal microbiota transfer (FMT) is the most drastic intervention to normalize a dysbiotic microbiota and now an accepted treatment for acute UC. Systematic reviews on randomized controlled trials demonstrate that FMT is effective in inducing clinical and endoscopic remission [5], being higher in patients who received FMT from multiple donors [6]. Both the donor and patient microbiota were important for treatment success [7]. Remission after FMT has been associated with butyrate producers, whereas relapse was associated with Proteobacteria [8]. More recently, bacterial strains associated with clinical response were identified [9] and stability of donor microbiota characterized as important for FMT success [10].

An important issue of FMT is safety, particularly in immunocompromised patients. The transfer of undefined microbiota entails uncontrollable risks for infection and other complications. Serious diseases caused by inadvertent transfer of pathogenic strains of *Escherichia coli* [11] and other bacteria prompted European Medicines Agency warnings in 2020. Therefore, it was investigated whether a sterile fecal microbiota filtrate (FMFT), containing phages, bacterial debris, metabolic products, and nucleic acids, could be used as an alternative strategy [12]. In a small cohort of five patients with recurrent *Clostridioides difficile* infection (rCDI) it was shown that FMFT was sufficient to eliminate symptoms, restore normal bowel habits, and change the gastrointestinal microbiota. Therefore, FMFT may represent an attractive approach for UC patients.

It is supposed that bacteriophages are the major players that regulate the composition and diversity of their bacterial hosts after FMFT [13]. In recent years metagenomic sequencing has significantly increased our knowledge on the human virome and the analysis of fecal viruses in healthy adults has revealed a high temporal stability of the human virome besides an individual specificity and correlation with the bacterial microbiome [14]. Several new databases have increased the described viral types by orders of magnitude [15–17] and allowed detailed studies including the analysis of virome changes associated with clinical conditions such as Metabolic Syndrome [18] or Inflammatory Bowel Disease (IBD) [19]. First studies on the gut virome in IBD were done by Norman et al. [20], where a later analysis of the same dataset could significantly improve the characterization of what has been known as viral dark matter [21]. This shows the importance of upgraded gut bacteriophage databases to reliably characterize the virome composition in FMFT donors and patients. However so far there are no studies describing changes of the patient virome during FMFT in UC patients. Accordingly, we developed a virome analysis pipeline and conducted the first case series examining the efficacy of FMFT and its effect on the microbial community and virome among patients with active UC.

## Materials and methods

### Patients, treatment regimes, and sample collection

**Figure 1:**
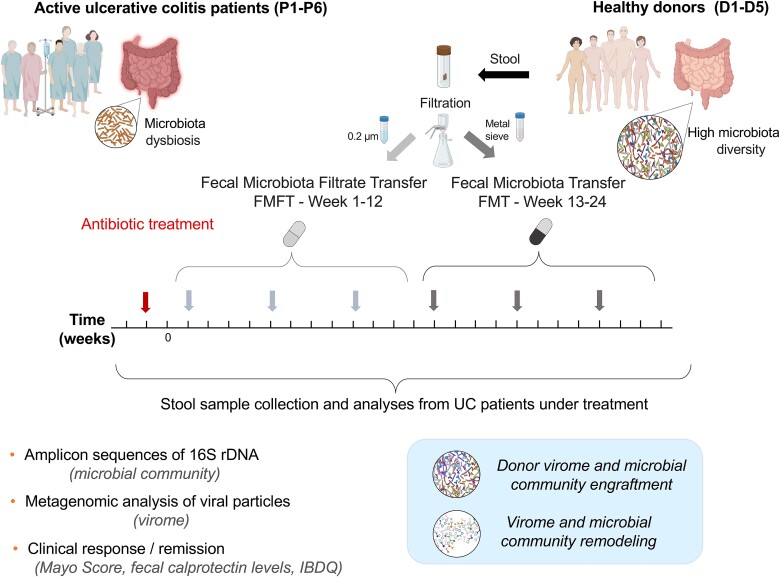
Study design. Six chronic UC patients P1–P6 (shown top left) received a preparatory antibiotic treatment. This was followed by FMFT (administered thrice in 12 weeks) and FMT (administered thrice in the following 12 weeks). Fecal microbiota and fecal microbiota filtrate were prepared from healthy donors D1–D5 (shown top right). Stool samples were collected during the treatment every week to track bacterial community changes using standard 16S rRNA gene amplicon sequencing and to determine the virome using as reference the Cenote Human Gut Virome Database. The health status of the patients was determined using standard clinical diagnostics.

We performed an open-label case series of long-term, multi-donor, oral-capsule based FMFT (12 weeks) followed by FMT (12 weeks) in patients with moderate to severe UC. Six adult patients with active disease and failure of response to conventional UC therapy were enrolled (Fig. 1). Patient characteristics are summarized in Supplementary Table S1.

The application of FMFT and FMT received full approval by the ethics committee of the Friedrich-Schiller-University Jena with reference numbers 4817-06/16 and 2021-2260-AMG-ff. All participants provided written informed consent.

The fecal microbiome was obtained from healthy donors who have been tested according to national and international recommendations [22]. Fresh donor stool was collected and stored in an airtight container at 4°C until processing (<2 hours after collection). Processing, capsule preparation and transfer to the patient were performed within 6 hours after collection (Supplementary Materials and methods).

Efficacy of FMFT/FMT was measured by clinical response (decrease in total Mayo score of >3 or > 30% at week 12/24) and clinical remission (a total Mayo score of 2, with no individual subscore exceeding 1, including the week 12/24 Mayo endoscopic score), fecal calprotectin level and Inflammatory Bowel Disease Questionnaire (IBDQ) values. Flexible sigmoidoscopy was performed before treatment and at week 12 and 24, respectively. Before FMFT/FMT patients were treated with vancomycin (four times 125 mg/day) and metronidazole (twice 400 mg/day) for 5 days (Fig. 1). This approach was based on previous observations suggesting that such a treatment could improve microbial engraftment in patients with UC [23, 24]. Afterwards, patients underwent FMFT (colonoscopic or nasojejunal application of 400 ml followed by twice 5 capsules per day for 5 consecutive days for 12 weeks, for deviations see Supplementary Table S1) followed by capsule FMT (twice 5 capsules per day for 5 consecutive days) for an additional 12 weeks. Multidonor FMFT and FMT was performed by application of different donor batches (Supplementary Table S1). Stool samples were collected before antibiosis (before treatment, bt), after antibiosis and during treatment for fecal calprotectin measurement and virome/microbiota analysis (Fig. 1 and Supplementary Table S2).

### DNA and 16S ribosomal RNA (rRNA) gene amplicon sequencing

DNA for 16S ribosomal RNA (rRNA) gene amplicon analyses was extracted from fecal samples using the FastDNA™SpinKit for Soil (MP Biomedicals, USA) comprising mechanical lysis using a Fast Prep®-24 (MP Biomedicals, USA). A 2-step PCR-approach was used to amplify the V1–V2 variable region of the 16S rRNA gene. PCR with primers 27Fbif and 338R containing part of the sequencing primer sites as short overhangs (given in italics; *ACGACGCTCTTCCGATCT*AGRGTTHGATYMTGGCTCAG and *GACGTGTGCTCTTCCGATCT*TGCTGCCTCCCGTAGGAGT, respectively) was used to enrich for target sequences (20 cycles). A second amplification step (10 cycles) added the two indices and Illumina adapters [25]. Amplified products were purified, normalized and pooled using the SequalPrep Normalization Plate (Invitrogen, Darmstadt, Germany) and subjected to 2 × 300-bp Illumina MiSeq sequencing (Illumina, San Diego, CA, USA).

### Bioinformatic and statistical analysis

The fastQ files were analyzed with the dada2 package version 1.21.0 in R [26]. Relative abundances of sequence types, species and genera were used for downstream analyses [27] (Supplementary Table S3). Diversity indices (species richness, Shannon diversity index H, Pielous evenness J) were calculated and multivariate analyses performed using PRIMER (v.7.0.11, PRIMER-E, Plymouth Marine Laboratory, UK), whereas univariate analyses were performed using Prism 9 (Graphpad Software, Inc.). Spearman correlations were calculated in Prism 9 based on bacterial species and virus type abundance matrices. Only virus types present at least once in an abundance >10% or thrice in an abundance >1% and bacterial species present at least once in an abundance >10%, five times in an abundance >1% or present in at least 15 samples analyzed were taken into account.

Differences in diversity indices between the time of FMFT and the time of FMT were tested for by unpaired *t*-tests. The data matrices comprising 667 species level taxa were used to construct sample-similarity matrices applying the Bray-Curtis algorithm, where samples were ordinated using non-metric multidimensional scaling (nMDS). The within-group homogeneity was tested by calculating multivariate dispersion indices with PRIMER. Centroids were calculated by permutational multivariate analysis of variance (PERMANOVA) based on species level Bray–Curtis similarity matrices and used to calculate similarities between treatments of patients (untreated, AB-treated, FMFT-treated, and FMT-treated) and donor samples.

### Fecal virome extraction, DNA purification, and sequencing

We designed a protocol to obtain viral DNA from stool samples for subsequent shotgun Illumina sequencing (Supplementary Materials and methods and Supplementary Table S4). Briefly, fecal material (300 mg) was diluted in 1.5 ml phosphate-buffered saline buffer and filtered through a 10 μm pore filter. The filtrate was washed, treated with DNaseI (Merck, Darmstadt, Germany), filtered through a 0.22 μm pore filter and concentrated in an Amicon ultra-15 filter device (Merck, Darmstadt, Germany). DNA from these concentrated bacteriophages was extracted using the GeneMATRIX Tissue and Bacterial DNA purification kit (EURX, Gdansk, Poland). An average of 3.5 μg of DNA was obtained when RNAlater had been applied to the sample either before storage or extraction. Libraries were constructed without size selection using the NEBNext Ultra II DNA FS Library Prep Kit (New England Biolabs, Frankfurt, Germany). Illumina NovaSeq sequencing of test stool samples yielded datasets of 16.3 10^6^ ± 0.7 10^6^ 250b paired-end reads per sample.

### Virome sequence analyses of donors and UC patients under FMFT and FMT treatment

Viromes were extracted from samples of six UC patients across the time of treatment and samples from five stool donors as well as from filtrate material used to prepare the FMFT solution. Datasets comprising 3.58 ± 0.06 Gb of sequence information were gathered per sample and used for mapping against the Cenote Human Virome Database (CHVD) [15] comprising 45,033 unique viral genomes from human gut metagenomic datasets (Supplementary Materials and methods). The resulting relative frequency matrix (Supplementary Table S5) was used to calculate Bray–Curtis similarities between samples.

Additional methods are provided in the Supplementary Materials and methods.

## Results

### Case histories and clinical outcomes

During the 12 weeks FMFT phase, no adverse events occurred in any of the six patients, except for mild complaints such as bloating and flatulence experienced by four patients within the first week. Importantly, patient 6 (P6) achieved clinical remission during FMFT (Mayo score decrease from 7 to 2 by week 12), while patients 1 and 3 (P1 and P3) showed clinical response (Mayo score drop from 8 to 4 and from 9 to 6, respectively; Table 1). These improvements were accompanied by a decrease in elevated fecal calprotectin levels and increase in IBDQ values (Table 1). Following the FMFT phase, five patients underwent an additional 12-week open FMT treatment period. After 24 weeks, P1, P3, and P6 achieved clinical remission, with P1 and P6 also achieving endoscopic remission (Mayo endoscopic subscore 1).

**Table 1:** Clinical characteristics and outcomes of patients who received FMFT and FMT.

	Patient 1	Patient 2	Patient 3	Patient 4	Patient 5	Patient 6
FMFT	12 weeks	12 weeks	12 weeks	12 weeks	12 weeks	12 weeks
Outcome week 12	Clinical response	No response	Clinical response	No response	No response	Remission
FMT	12 weeks	12 weeks	12 weeks	4 weeks ^a^	12 weeks	12 weeks
Outcome week 24	Remission	No response	Clinical response	No response	No response	Remission
Mayo endoscopic subscore						
Baseline	2	2	3	3	3	3
Week 12	1	2	3	3	3	1
week 24	1	3	2	NA	3	1
Fecal calprotectin						
Baseline	1151	>2000	581	884	1880	480
Week 12	105	>2000	160	719	>2000	66.6
Week 24	200	>2000	86.7	NA	>2000	1990
IBDQ						
Baseline	85	79	119	131	196	112
Week 12	176	70	166	125	136	187
Week 24	169	92	153	NA	162	204

a Patient 4 terminated at own request after 4 weeks.

NA, not available.

### Bacterial diversity and community structure during FMFT and FMT

Donor bacterial communities were highly diverse (high richness S, evenness J, and Shannon diversity H) with species richness rarely falling <100 (Supplementary Table S6). Antibiotic treatment resulted in vast changes in the bacterial communities with Bray–Curtis similarities compared to pre-treatment communities <10.5% in 5 of 6 patients. Also, community diversity measures decreased substantially (Supplementary Figs S1 and S2). During the time of FMFT, bacterial microbiota did not completely recover from antibiotic triggered diversity reduction and diversity was just restored by FMT (Supplementary Fig. S2).

**Figure 2:**
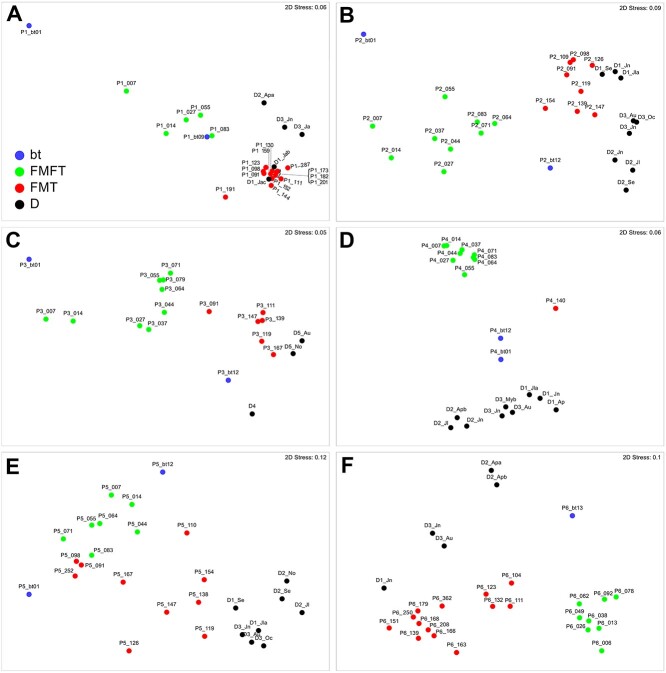
Differences in global bacterial community structures in patients during FMFT and FMT. The global bacterial community structures in patients P1 (A), P2 (B), P3 (C), P4 (D), P5 (E), and P6 (F) during FMFT (red dots) and FMT (green dots) were assessed by nMDS and are based on standardized species abundance data. Similarities were calculated using the Bray–Curtis similarity algorithm. The treatment time (in days) is indicated relative to the start of treatment (first FMFT), with bt indicating days before treatment. The antibiotic treated sample of P6 is not shown as its low similarity prevented a meaningful visualization.

P1 showed clinical response during FMFT and remission during FMT. Upon FMFT the bacterial community after AB treatment (Com_AB_) returned to a structure similar to that before AB treatment (Com_BT_; distance among centroids [dac] 42%), but distant to that of the FMFT donor 1 (D1, dac = 68%; Fig. 2A and Supplementary Fig. S3). Such a return in community structure was unique among the patients. Upon FMT, the community after FMFT (Com_FMFT_) became highly similar to that of D1 (dac = 26%, Supplementary Table S7). The multivariate dispersion, which was 1.247 for Com_FMFT_ was reduced to 0.607 for Com_FMT_ indicating a highly stable community after FMT (Supplementary Table S8), which remained stable even 140 days after the last FMT. In patient 2 (P2), who did not respond to treatment, the community after multidonor FMFT, Com_FMFT_, adopted a structure dissimilar to both Com_BT_ and any of the donor communities (dac >65%; Fig. 2B). FMT resulted in a structure with similarities to D1 (dac = 40%). Higher multivariate dispersions were observed compared to P1 (Supplementary Table S8). Similarly, the Com_FMFT_ community of P3 who showed clinical response already during FMFT adopted a new relatively stable state, highly dissimilar to Com_BT_ as well as any of the donor communities (dac >76%; Fig. 2C). Multidonor FMT resulted in an increase in similarity of the communities to D5 (dac = 44%). P4 who showed no clinical response during FMFT was the only patient where antibiotic treatment was ineffective and the dissimilarity between communities before and after treatment was only 31% contrasting dissimilarities of >90% in all other patients (Fig. 2D). Like in P2 and P3, the Com_FMFT_ community adopted a stable state highly dissimilar to Com_BT_ as well as any donor community (dac >71%, multivariate dispersion of Com_BT_ = 0.658). Only one sample was available after FMT such that the effect of that treatment could not be evaluated. In contrast to all other patients, communities of P5 (no clinical response during treatment), both after multidonor FMFT and FMT were highly disperse (multivariate dispersion of 1.648 and 1.68 respectively), indicating that no stable communities were formed. There were no indications of successful transfer of bacteria (Fig. 2E). These results contrast those observed in P6 (remission already during FMFT), where Com_FMFT_ resulted in a highly stable (multivariate dispersion of 0.558) community distinct to that of Com_BT_ (Fig. 2F). FMT led to a community with increased similarity to donor D1 and D3 (dac = 40% and 42%, respectively), which remained stable even 210 days after the final third FMT. Overall, FMFT typically resulted in stable bacterial communities distinct to those of donors and recipient, whereas FMT typically provokes the formation of communities with similarity to at least one of the donors.

**Figure 3:**
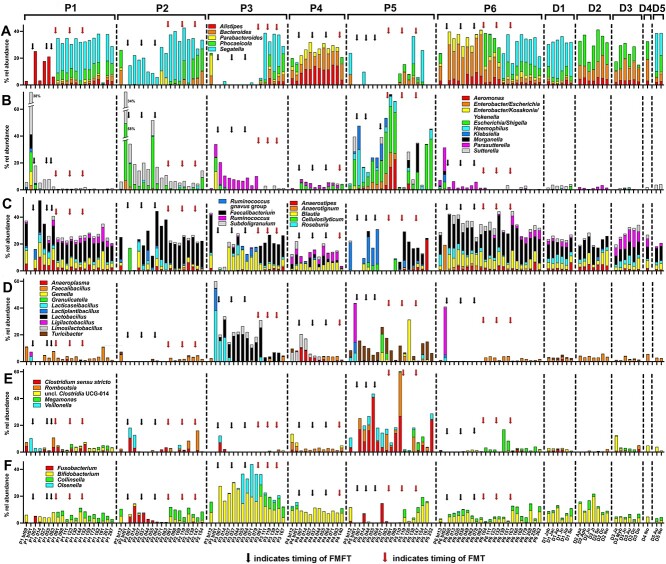
Relative abundance of genera and genus level taxa in patients during treatment as well as in donors. The abundance of members of the following higher-level taxa are shown: (A) Bacteroidales, (B) Proteobacteria, (C) *Lachnospiraceae* and *Ruminococcaceae*, (D) Bacilli, (E) other Firmicutes, and (F) *Fusobacterium* and Actinobacteriota. Only genera and genus level taxa present at least once in a relative abundance >10% are visualized. The time of sampling in patients (days) is shown to the bottom with bt indicating the sample before treatment. The origin of the samples is indicated to the top (patients P1–P6 or donors D1–D5).

The success of FMT to modulate the patient bacterial community was supported by the establishment of genera previously absent or below the detection level (Fig. 3A–F). For example, out of the Bacteroidota phylum, only *Alistipes* was present in high relative abundance in P1 (2.8%–8.1%) and regained abundance (up to 25%) after FMFT (Fig. 3A). However, only after FMT *Parabacteroides* appeared at relative abundances of 1.03 ± 0.15 SEM % and all *Bacteroides*, *Phocaeicola*, and *Segatella* increased from mean abundances <0.3% before FMFT to mean abundances of 1.87 ± 0.30%, 6.8 ± 0.8% and 19.9 ± 2.0%, respectively, after FMT. There were no indications that any of the abundant donor genera were influenced in abundance after FMFT. However, the original host community recovers, as indicated by depletion of *Enterobacteriaceae* and recovery of *Faecalibacterium* (Fig. 3B and C).

**Figure 4:**
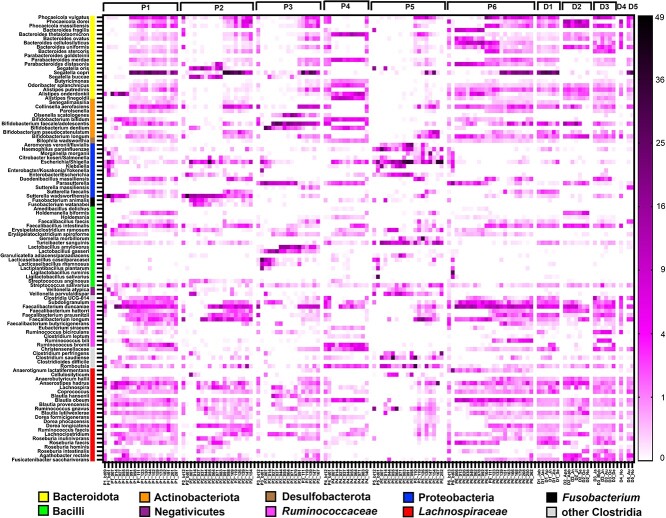
Heatmap showing the relative abundance of species level taxa in patients and donors during treatment time. Only taxa of high abundance (at least once in a relative abundance >5%) or reported as of importance in UC are shown. The scale (% relative abundance) is given to the right.

P2 is exceptional among patients, as *Segatella*, present in the patient community only in low abundance gained a relative abundance of 15.0 ± 1.9% after FMFT (Fig. 3A). However, this is due to an increase of *S. buccae* and *S. oris* (Fig. 4), which both were practically absent from all donor communities. *S. copri* increased to high levels only after FMT. Similarly, *Fusobacterium*, the abundance of which never exceeded 0.1% in any donor sample reached 4.2 ± 1.3% during FMFT, likely due to recovering and remodeling of the original bacterial community. As in P1, *Bacteroides, Phocaeicola*, and *Segatella* increased in abundance only after FMT.

AB treatment in P3 resulted in an increase in *Lactobacillaceae*, which decreased only after successive FMT (Fig. 3D). Both Bacteroidota and *Faecalibacterium* remained underrepresented during FMFT and only recovered after successive FMT.

Whereas no tremendous changes in higher taxa were evident in P4 (only one sample after FMT was available), no stable community pattern could be reached during FMFT and FMT in P5. *Enterobacteriaceae* and *Clostridium sensu stricto* remained important community members and neither *Faecalibacterium* nor Bacteroidota could reach a stable share of the community even after FMT (Fig. 3A, B, C, and E).

In P6, as in P3 and P5, *Lactobacillaceae* account for a dominant proportion of the microbial community after AB treatment. However, they rapidly disappear concomitantly with FMFT with *Bacteroides*, *Alistipes*, and *Parabacteroides*, becoming dominant members as they have been before FMFT, indicating mainly a recovering of the original community (Fig. 3A and D).

Clearly during FMFT, microbial communities adopted a stable composition in five out of six patients, which was distinct from the original patient microbial community in four cases. However, a change of the community to one similar to a donor community was not observed. In contrast, FMT clearly changed the community to a microbiota with similarity to at least one of the donors in four of five patients followed.

### Bacterial taxa of specific importance in UC

Patients with UC are reported to harbor a dysbiotic community characterized by increased levels of *Ruminococcus gnavus* [4] or *Haemophilus parainfluenzae* [3] and decreased levels of SCFA producers [3]. Additionally a set of taxa has been identified as important donor bacteria associated with treatment success (*Odoribacter splanchnicus* [9], *Anaerobutyricum [Eubacterium] hallii*, *Parabacteroides merdae* [10]) or failure (*Sutterella*). Similar trends were also observed here, with beneficial bacteria of higher abundance in donors (Fig. 4).

Importantly, the remodeling of the communities during FMFT was sufficient to allow a clinical response of three patients. In P1, depletion of *Clostridioides difficile* may be a major reason for recovery. *O. splanchnicus* [9] was present at all time points in all five donors, but also in three patients (Fig. 4). A reaction after FMFT was only seen in the not recovering P4, where *O. splanchnicus* abundance increased after AB treatment and then rapidly declined during FMT (Fig. 4). Beneficial bacteria such as *Agathobacter rectale* [2] or *Anaerobutyricum hallii* were present in most of the donors, but were not enriched in any patient before FMT (Fig. 4). However, enormous community restructuring was evident during FMFT. This is most obvious in P4 and P6 where *Blautia obeum* increased by 1–2 orders of magnitude in relative abundance compared to pre-AB treatment levels (Fig. 4**)**. *Veillionella parvula/dispar* reached 3.8%–9.2% relative abundance during early FMFT treatment times of P1, P2, and P3 (Fig. 4).

### Virome in donors and patients

**Figure 5:**
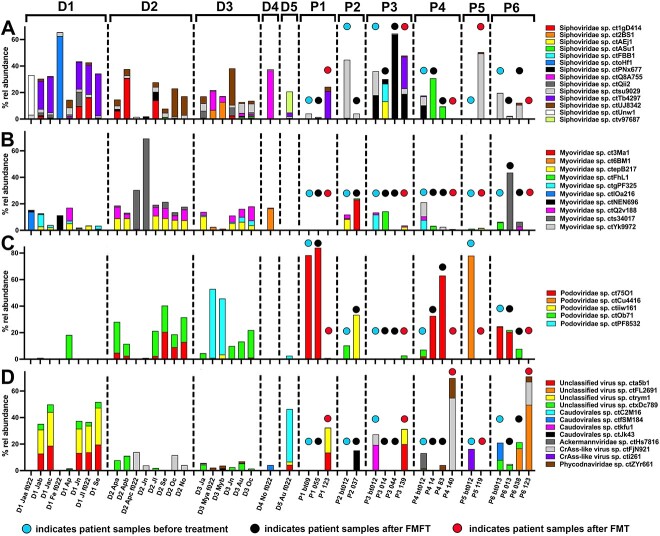
Relative abundance of virus particles in the virome of donors and patients: (A) Siphovididae, (B) Myoviridae, (C) Podoviridae, and (D) other viruses. Only particles with a relative abundance >10% in at least one sample are shown. The month of donor sampling in 2019 is indicated by a 2–3-letter code. Filtrates were prepared either via a standard protocol or a protocol including an additional initial filtering through a 0.22 μm pore filter (f022) designed for application to patients. The time of sampling in patients (days) is shown to the bottom with bt indicating the sample before treatment . Samples after 37–55 days were collected after FMFT and samples after 119–140 days after FMT. The origin of the samples is indicated to the top (donors D1–D5 or patients P1–P6).

Analysis of viromes of donor samples collected over time in 2019 revealed Bray–Curtis similarities >68% for D1 (5 of 8 samples), >46% for D2 (6 of 8 samples) or > 52% for D3 (4 of 6 samples) indicating a high stability. However, some samples were quite distinct indicating also some variability of the virome over time (Fig. 5A–D). Comparison of filtrates produced from the same input donor stool sample using (i) a fast protocol necessitating low input material developed here and (ii) filtrates from donor samples, which were used for FMFT capsule preparation (Supplementary Materials and methods) showed that these methods can produce similar results (see D3_Mya_f022 and D3_Myb which share 74% similarity; Supplementary Table S9). However, some filtrates were quite distinct (see D2-Apc_f022) and may constitute different virome states (Fig. 5).

From all patients, samples before treatment, two samples during FMFT and one sample after FMT were analyzed for viral content. The corrected read count was 316,793 ± 151,668 for patient samples and 249,287 ± 22,221 for donor samples. In five of the 24 patient samples the count was <1000 and thus no reliable virome analysis was possible. These cases included three samples 14 days after treatment start where possibly a virome has not yet been recovered after AB treatment (Supplementary Table S5).

**Figure 6:**
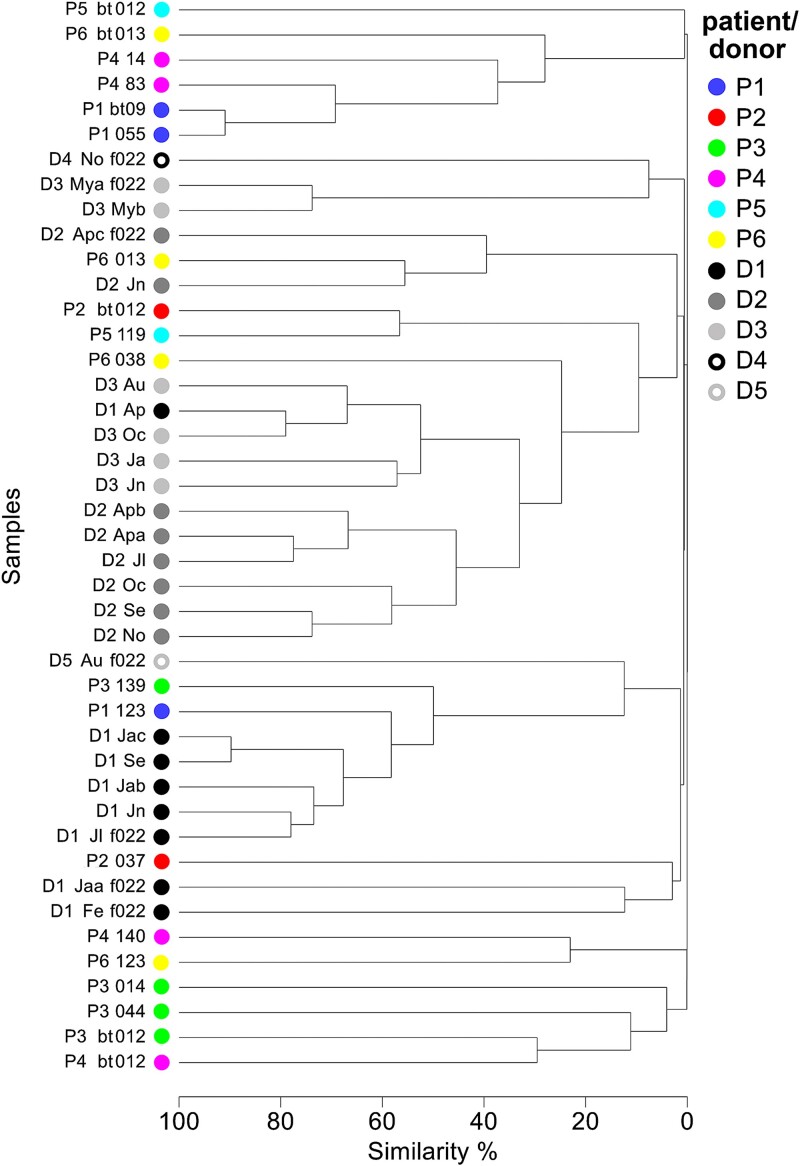
Similarities in global virome structures of patients and donors. The Bray–Curtis similarities in global virome structures in patients P1–P6 and donors D1–D5 were assessed by complete linkage clustering and are based on relative abundance data. The treatment time (in days) of patients is indicated relative to the start of treatment (first FMFT), with bt indicating days before treatment. The month of donor sampling in 2019 is indicated by a 2–3-letter code. Filtrates were prepared either via a standard protocol or a protocol including an additional initial filtering through a 0.22 μm pore filter (f022) designed for application to patients.

In P1 the virome after the second FMFT showed a high similarity to the original virome, indicating its recovery after AB treatment (Fig. 6 and Supplementary Fig. S4). This similarity was mainly due to the high abundance of *Podoviridae* sp. ct75O1 accounting for 78% and 84% of viral particles before treatment and after the 2^nd^ FMFT, respectively (Fig. 5C). Other viruses abundant in P1_bt_ were also observed after FMFT. These results are in accordance with the bacterial community analysis where high similarity was observed between the community before and after FMFT. FMT completely altered the virome (as it changed the bacterial community) and high similarity (73%–78%) was observed to the main virome state of D1 (Fig. 6 and Supplementary Fig. S4). This is due to the high abundance of *Siphoviridae* sp. ctTb4297 and two unclassified viruses (ctrym1 and cta5b1) for all of which *S. copri* is the predicted host (Fig. 5A and D, Table 2, and Supplementary Table S10). This virome is in agreement with the establishment of part of the D1 microbiota after FMT in P1.

**Table 2:** Predicted bacterial hosts of main viruses in the viromes of patients and donors.

Virus ID CHVD	CRISPR spacer-based bacterial host prediction^a^	BLASTN host prediction^b^	Correlation with^c^	Rho
*Ackermannviridae* sp. ctHs7816	*Niabella*	*Barnesiella intestinihominis*		<0.6
*Caudovirales* sp. ctC2M16	No host detected	*Prevotella copri*		<0.6
*Caudovirales* sp. ctfSM184	*Blautia*	*Dorea longicatena*	*Faecalibacterium butyricigenerans*	0.702
*Caudovirales* sp. ctJk43	*Sutterella*	*Pectobacterium atrosepticum*		<0.6
*Caudovirales* sp. ctkfu1	*Flavonifractor*	*Clostridiales bacterium*		<0.6
CrAss-like virus sp. ctFjN921	*Eubacterium*	*Bacteroides xylanisolvens*	*Alistipes finegoldii*	0.623
CrAss-like virus sp. cti261	*Prevotellaceae*	*Bacteroides uniformis*		<0.6
*Myoviridae* sp. ct3Ma1	No host detected	*Erwinia amylovora*		<0.6
*Myoviridae* sp. ct6BM1	*Listeria*	*Ruminococcus bicirculans*	*Ruminococcus bicirculans*	0.867
*Myoviridae* sp. ctepB217	*Bacteroides*	*Bacteroides cellulosilyticus*	*B. xylanisolvens*	0.644
*Myoviridae* sp. ctFhL1	*Salmonella*	*Escherichia coli*	*E. coli*	0.822
*Myoviridae* sp. ctgPF325	*Ruminococcaceae*	*Faecalibacterium prausnitzii*	*F. prausnitzii*	0.775
*Myoviridae* sp. ctlOa216	Unknown	*Intestinimonas butyriciproducens*	*Sutterella faecalis*	0.746
*Myoviridae* sp. ctNEN696	No host detected	*F. prausnitzii*	*Enterorhabdus*	0.654
*Myoviridae* sp. ctQ2v188	*Bacteroides*	*Bacteroides fragilis*	*B. uniformis*	0.754
*Myoviridae* sp. cts34017	*Bacteroides*	*B. xylanisolvens*	*Bacteroides zhangwenhongii*	0.694
*Myoviridae* sp. ctYk9972	*Thermoanaerobacterium*	*Ruminococcus bromii*	*R. bromii*	0.665
*Myoviridae* sp. ct6ca219	*Bacteroides*	*Bacteroides dorei*	*Paras. excrementihominis*	0.650
*Myoviridae* sp. ct7fO419	*Bacteroides*	*Bacteroides vulgatus*	*B. xylanisolvens*	0.722
*Myoviridae* sp. ctITS746	*Prevotella*	*Parabacteroides distasonis*	*Lachnoclostridium edouardi*	0.660
*Phycodnaviridae* sp. ctZYr661	*Alistipes*	*Fusicatenibacter saccharivorans*		<0.6
*Podoviridae* sp. ct75O1	No host detected	*Alistipes ihumii*	*Alistipes onderdonkii*	0.769
*Podoviridae* sp. ctCu4416	*Bacteroides*	*B. uniformis*		<0.6
*Podoviridae* sp. ctiiw161	*Sutterella*	*Dickeya solani*		<0.6
*Podoviridae* sp. ctOb71	No host detected	*B. uniformis*	*B. uniformis*	0.659
*Podoviridae* sp. ctPF8532	No host detected	Salmonella enterica*		<0.6
*Siphoviridae* sp. ct1gD414	*Lachnospira*	*Lachnospira eligens*	*Lachnospira rugusae*	0.711
*Siphoviridae* sp. ct2BS1	Clostridiales	*Ruminococcus bicirculans*	*Vescimonas*	0.835
*Siphoviridae* sp. ctAEj1	*Bifidobacterium*	*Bif. pseudocatenulatum*		<0.6
*Siphoviridae* sp. ctASu1	No host detected	*Alistipes ihumii*	*A. finegoldii*	0.748
*Siphoviridae* sp. ctFBB1	*Bifidobacterium*	*Bif. pseudocatenulatum*	*Bifidobacterium dentium*	0.658
*Siphoviridae* sp. ctoHf1	*Lachnospiraceae*	*F. prausnitzii*	*Vescimonas*	0.656
*Siphoviridae* sp. ctPNx677	*Bifidobacterium*	*Bifidobacterium longum*		<0.6
*Siphoviridae* sp. ctQ8A755	*Ruminococcus*	*Ruminococcus bicirculans*	*Ruminococcus bicirculans*	0.864
*Siphoviridae* sp. ctQii2	*Roseburia*	*Roseburia inulinivorans*	*F. prausnitzii*	0.611
*Siphoviridae* sp. ctsu9029	*Anaeromassilibacillus*	*F. prausnitzii*		<0.6
*Siphoviridae* sp. ctTb4297	*Prevotella*	*P. copri*	*Segatella copri*	0.691
*Siphoviridae* sp. ctUJ8342	*Bacteroides*	*Bacteroides salyersiae*	*B. xylanisolvens*	0.607
*Siphoviridae* sp. ctUnw1	*Tessaracoccus*	*Clostridium phoceensis*	*Senegalissima*	0.761
*Siphoviridae* sp. ctv97687	*Dorea*	*Flavonifractor plautii*	*B. intestinihominis*	0.688
*Siphoviridae* sp. ct3cx1	*Blautia*	*Ruminococcus faecis*	*Ruminococcus gnavus*	0.695
*Siphoviridae* sp. ctBeZ167	*Prevotellaceae*	*Parabacteroides merdae*		<0.6
*Siphoviridae* sp. ctmIY117	*Bacteroides*	*P. distasonis*		<0.6
*Siphoviridae* sp. ctpHQ1	*Alistipes*	*Alistipes shahii*		<0.6
*Siphoviridae* sp. ctrxw1	No host detected	*Fusobacterium nucleatum*		<0.6
*Siphoviridae* sp. ctZDc254	*Bacteroides*	*P. distasonis*	*B. xylanisolvens*	0.743
*Siphoviridae* sp. ctZgP182	*Bacteroides*	*P. distasonis*	*B. uniformis*	0.673
Unclassified virus sp. cta5b1	No host detected	*P. copri*	*Segatella copri*	0.692
Unclassified virus sp. ctFL2691	*Pectobacterium*	*P. copri*		<0.6
Unclassified virus sp. ctrym1	*Prevotella*	*P. copri*	*Segatella copri*	0.686
Unclassified virus sp. ctxDc789	*Roseburia*	*R. inulinivorans*	*Roseburia faecis*	0.822

a CRISPR spacer-based bacterial host predictions were performed using CrisprOpenDB [56].

b BLASTN host prediction was performed in PhageScope [57] and by DeepHost [34]

c Pairwise spearman correlations between virus type and bacterial species abundances were calculated in Primer9. Only correlations of rho >0.6 are given. In case of multiple correlations of rho >0.6 only the correlation with the highest rho is given.

The three mentioned *S. copri* viruses were also observed in P3 after FMT using D4 and D5 as donors. In fact, they were present in reasonable abundances (2.4%–4.3%) in D5 which has been indicated as a successful bacterial community donor. However, the abundant *Caudovirales* sp. ctC2M16, probably also a *S. copri* phage (Fig. 5D), and *Siphoviridae* sp. ctv97687 (Fig. 5A), were not transferred in substantial amounts and the overall similarity between the virome of P3 after FMT and D5 reached only 15%. Overall, the virome in P3 was quite variable with *Siphoviridae* sp. ctPNx677, a putative *Bifidobacterium* phage, being observed throughout the whole treatment time and dominating the virome after the second FMFT (Fig. 5A). There was no evidence for establishment of viral particles from D4 or D5 during FMFT but obviously a remodeling of the virome (Fig. 6 and Supplementary Fig. S4).

*Podoviridae* sp. ct75O1, a predicted *Alistipes* phage was not only an important constituent of the P1 virome, but was also detected in P4 and P6 before treatment (Fig. 5C). In accordance with the relative abundance of *Alistipes* in those patients, this virus remained in both patients during the FMFT phase and disappeared after FMT. In both patients after FMT the circular CrAss like virus sp. ctFjN921 became abundant (5% and 17% relative abundance, respectively), putatively originating from D2 where this phage was an important virome member (Fig. 5D). In P6 also the circular unclassified virus sp ctFL2691 was of high abundance (50%). However, this virus was already present during the FMFT phase but not in any of the donors and thus a component of the virome remodeling in P6 (Fig. 5D). In both P4 and P6 a severe remodeling of the virome could be observed, without any clear evidence of virome transfer from any donor. Also in P2, a remodeling of the virome during FMFT was visible with the proteobacterial phages *Podoviridae* sp. ctiiw161 and *Myoviridae* sp. ct3Ma1 dominating the community 37 days after treatment start. Both were absent from the patient virome (Fig. 5B and C). However, ctiiw161 was present in the D3 virome such that an establishment cannot be excluded.

Overall, the analysis gave indications of severe virome changes during FMFT with establishment of donor viruses being of minor importance whereas during FMT an establishment of donor viruses could be evidenced.

## Discussion

This open-label case series of FMFT followed by FMT in patients with moderate to severe UC suggests that FMFT and FMT are safe treatment options and should be evaluated in a clinical trial in comparison with placebo. A key advantage of FMFT is the avoidance of risks inherent to the transfer of bacterial communities. Although the clinical treatment results in this case series should not be overestimated, three out of six patients showed clinical improvement already during FMFT (Supplementary Table S6). It is unclear whether this improvement is solely attributable to the FMFT or if antibiotic pretreatment also played a role. Against this background, the FRESCO study (Transfer of FRozen Encapsulated Multidonor Stool Filtrate for Active Ulcerative COlitis; NCT03843385) that is currently recruiting, aims to clarify this question [28].

The most common phages of the gut had previously been classified as belonging to the families *Myoviridae*, *Podoviridae*, and *Siphoviridae* of the order *Caudovirales* [14]. We still refer to this historical classification to facilitate comparisons to previous findings. As these families are not monophyletic [29] they were abolished recently and replaced by the class *Caudoviricetes* [30]. These phages are also the most abundant phages identified here. In order to compare samples, we relied on genome sequence similarity to define clusters of virus variants mapping to genome references of the CHVD gut viral genomes dataset [15]. However, a comprehensive viral taxonomy reflecting evolutionary relatedness is still to be established [31].

*Crassvirales* of the *Caudoviricetes*, previously identified in the majority of human gut metagenomes [32], were also prevalent in our study. Besides, we detected *Ackermannviridae*, *Herelleviridae*, and *Inoviridiae*. It has been reported that IBD patients show viral imbalances characterized by a high relative abundance of *Caudovirales* [33] and a low relative abundance of *Microviridae* phages [19]; however, due to the improving capability to better characterize the “viral dark matter”, such generalizations need to be taken with care. Interestingly *Microviridiae* were nearly omnipresent here. However, they never exceeded a relative abundance of 0.5% and the low amount of patients and donors prevent any association study.

We could show that the virome experienced enormous changes during both FMFT and FMT. Changes during FMT were correlated with transfer of the predicted host as evidenced by the increase in relative abundance of phages probably hosted by *Segatella* in all samples where an establishment of *S. copri* could be determined. Overall, there was a high correlation (rho >0.7) between the relative abundance of the host predicted by DeepHost [34] and the abundance of a phage for ~40% (Table 2) of the most abundant and prevalent phages detected here. It is, therefore, reasonable to assume that various phages transferred during FMFT could not establish in patients as their bacterial host was not available. However, even though prediction of hosts has improved significantly by analyzing for bacterial CRISPR spacer sequences with homology to known viral genome sequences or by analyzing integrated prophage sequences, there is still a gap for universal tools [35]. Also negative correlations between a phage and its predicted host have been described, even though only a few were evident here (Supplementary Table S11). As an example, a higher abundance of *Faecalibacterium* phages in IBD patients compared to healthy controls has been reported, even though patients harbored a lower abundance of *Faecalibacterium* indicating that these phages are activated during disease and trigger *Faecalibacterium* depletion [36]. Similarly, *Klebsiella* spp., overrepresented in UC and inducing a pro-inflammatory response in mice, is targeted by specialized phages [37] resulting in effective *Klebsiella* suppression and attenuated inflammation. An expansion of *Klebsiella* phages after FMT in humans has also been correlated recently with a concordant decrease of *Klebsiella* spp. and striking increase of *Escherichia* phages [38].

Bacteriophage predation does not only affect susceptible bacteria but also induces disturbances to other bacteria via interbacterial interactions [39], modulating the gut metabolome, triggering immunomodulatory effects. Also phages themselves have an immune modulatory effect in diseases such as IBD and differences in diversity and abundance of bacteriophages proliferating in the human gut microbiome can result in distinct immunogenic responses [40]. Furthermore, phages can adhere to mucus layers thereby reducing microbial colonization [41], may alter mucosal immunity impacting mammalian health [42] and may even pass the intestinal epithelium to directly interact with the enteric immune system [43]. However, our knowledge how complex phage communities impact bacterial communities is still limited and further studies on those interactions are required.

The first study on FMFT involved five patients suffering from rCDI. All patients responded to treatment [12] and community changes were visible, similar with our findings, where FMFT after antibiotic treatment typically resulted in stable microbial communities distinct from those originally present or present after antibiotic treatment. However, it should be noted that controls assessing microbiota changes after antibiotic treatment without FMFT have not been performed in these patient groups. Interestingly the decrease in *Enterobacteriaceae* abundance was discussed as one possible reason of FMFT treatment success and it was suggested that FMFT may be applicable to other diseases with *Enterobacteriaceae*-driven dysbiosis such as IBD [12]. We could note here that antibiotic treatment resulted in a transient increase in *Enterobacteriaceae* abundance which was reversed during FMFT treatment. If the decrease in *Enterobacteriaceae* is actually caused by FMFT or a recovery of the community remains to be elucidated. The authors also claimed the virome to be significantly changed and being dominated by *Lactococcus* phages [12]. However, *Lactococcus* is not an important member neither of the microbiota of any donor or recipient nor of the gut microbiota in general, indicating that those virome results needs to be reevaluated given the extensively improved representation of gut bacteriophage genomes in public databases.

The change in the virome upon FMT in rCDI patients has been investigated by various authors. A long-lasting remodeling of the virome to one resembling the donor was observed [44] or the microbial community was restored to a healthy community where e.g. Proteobacteria, but also virome disbalances were eliminated [45, 46]. Fecal viral transfer has since then been identified as important to reshape microbial communities after antibiotic treatment [47], to drive lean and obese phenotypes in mice [48], to prevent necrotizing enterocolitis in piglets [49] or even to reduce symptoms of type 2 diabetes in mice [50]. Moreover a small study comparing the use of FMFT versus FMT in rCDI observed comparable remission rates [51].

Our study revealed remission of one patient and clinical response of two patients already after FMFT. This is in accordance with the observed virome and microbial community response, which both changed enormously under FMFT. No control group was included in this hypothesis-generating preliminary study. We cannot therefore rule out that community remodeling is not due to FMFT but to recovery of the communities after antibiotic treatment. However, the effect of antibiotic treatment on human gut communities has been subject to various studies and it is generally accepted that the gut microbiota is significantly perturbed by antibiotic use, but recovering to a community closely resembling its pretreatment several weeks after exposure [52, 53]. Even though it has to be noted that recovery of the microbial communities can be variable [54] importantly only one of the patient communities analyzed here returned to a community with similarity to the pretreatment state after antibiotic exposure as evidenced by the dac. A recent study on FMFT transfer in patients with metabolic syndrome [55] showed that treatment produced changes very different to those observed after treatment with placebo. Moreover, virus particles transferred from UC patients and controls to mice induced inflammation together with alterations in the gut virome and bacterial community [13] evidencing the virome to be an important constituent for disease.

## Supplementary Material

Supplementary_Methods_and_Figures_ycae167

Supplementary_Tables_ycae167

## Data Availability

Amplicon sequence data are available at the NCBI Sequence Reads Archive under BioProject accession number PRJNA11057508 https://dataview.ncbi.nlm.nih.gov/object/PRJNA1105750?reviewer=dd7bfoc4smtk1114sr7oiduiln) and raw sequence datasets of virome samples at the Harvard Dataverse project https://dataverse.harvard.edu/privateurl.xhtml?token=f54def50-8f5d-4b83-b1f4-1535a089bd52.
