# Supplementary material for: Bacterial and viral assemblages in ulcerative colitis patients following fecal microbiota and fecal filtrate transfer
Source: ISME Commun. 2024 Dec 23;5(1):ycae167. doi: 10.1093/ismeco/ycae167 (PMC11740987; doi:10.1093/ismeco/ycae167)
Supplement: Supplementary_Methods_and_Figures_ycae167 [file supplementary_methods_and_figures_ycae167.pdf]

Supplementary methods and figures to

Bacterial and viral assemblages in ulcerative colitis patients following fecal microbiota and fecal filtrate transfer

Short title: Virome upon fecal (filtrate) transfer

Howard Junca[1*], Arndt Steube[2*], Simon Mrowietz[2], Johannes Stallhofer[2], Marius Vital[1], Luiz Gustavo dos Anjos Borges[1], Dietmar H. Pieper[1*] and Andreas Stallmach[2*]

[1]Microbial Interactions and Processes Research Group Helmholtz Centre for Infection Research, Braunschweig, Germany

[2]Department of Internal Medicine IV (Gastroenterology, Hepatology and Infectious Diseases) University Hospital Jena, Jena, Germany.

*contributed equally

Correspondence: Dietmar H. Pieper, Microbial Interactions and Processes, Helmholtz Centre for Infection Research, Inhoffenstr. 7, D 38124 Braunschweig, Germany. e-mail: dpi@helmholtz-hzi.de

This supplement contains supplementary methods and supplementary figures S1-S4.

Supplementary tables S1-S11 are provided in a separate Excel file.

Overview on supplementary information:

Supplementary Methods, Supplementary Materials and Methods

Supplementary Figure S1, Bacterial community diversity in patients P1 - P6 during treatment time.

Supplementary Figure S2, Bacterial community diversity in patients P1 - P6 before (bt), during FMFT (f) and during FMT (m).

Supplementary Figure S3, Differences in global bacterial community structures.

Supplementary Figure S4, Differences in global virome structures of patients and donors.

Supplementary Materials and Methods

Preparation of donor stool and stool filtrate for transfer

Donor stool for FMT was prepared under ambient air and basically as previously described [1]. Donor stool (70–250 g) was mixed with 2 ml sterile 0.9% NaCl per 1 g stool, homogenized, and filtered through a metal sieve to eliminate solid particles. Subsequently, the material was mixed with 10% glycerol, double encapsulated in enteric capsules (Vcaps enteric™, size 0 and 00, Capsugel/Lonza) and frozen at -80 °C.

The procedure for preparation of stool filtrate was adopted from Ott et al. [2] For FMFT, 100 g donor stool was mixed with 300 ml sterile 0.9% NaCl, homogenized and filtered through a metal sieve. The suspension was centrifuged at 3000 g for 30 min to sediment solid components, the supernatant was collected and filled up to 1000 ml with 0.9% NaCl. The suspension was then filtered under compressed air (depth filter 0.5-15.0 µm and EKV filter SUPOR EKV filter 0.2 µm). Subsequently, 400 ml filtrate was applied via coloscopic or nasojejunal transfer to patients (**Supplementary Table 1**). Further filtrate was mixed with 10% glycerol, double encapsulated in enteric capsules (Vcaps enteric™, size 0 and 00, Capsugel/Lonza) and frozen at -80 °C. Stool from five donors was processed for the production of FMFT and FMT. Filtrates and donor stool (dissolved in sterile saline) were streaked on sterile blood agar plates (Columbia agar with 5% sheep blood) and cultured anaerobically and aerobically at 37°C for up to 48 hours at the Institute of Medical Microbiology, Jena University Hospital, Jena, Germany. No bacterial growth was observed from filtrates, while donor stool showed abundant growth under both conditions.

Amplicon sequence variants generation and annotation

The quality-trimming of fastQ files and filtering steps were performed using the filterAndTrim function. Forward and reverse reads were trimmed on the 5'-end by 20 and 19 bases, respectively. Reads were truncated to a length of 240 bases and a maximum of 2 expected errors per read was permitted. After denoising and paired-end reads merging, chimeras were removed. Remaining non-bacterial sequences (eukaryota, mitochondria, chloroplast) were manually deleted. Overall, 5.356.963 bacterial 16S rDNA sequence counts were obtained with a mean of 40.583 ± 9104 reads per sample (**Supplementary Table 3a** at https://www.doi.org/10.5281/zenodo.13221182). Sequence types were annotated based on the naïve Bayesian classification with a pseudo-bootstrap threshold of 80% using RDP set18[3] (**Supplementary Table 3c** at https://www.doi.org/10.5281/zenodo.13221182). Sequence variants were then manually analyzed against the RDP database using the Seqmatch function to define the discriminatory power of each sequence type. All annotations were then upgraded using SILVA SSU138.1 rRNA database[4]. In some cases where genus

or higher level annotations were distinct between SILVA SSU138 on the one hand and RDP and the LPSN database [5] on the other hand LPSN annotations were used (e.g. differentiation of *Lactobacillus* or *Prevotella* in different genera). In addition to sequences with species level annotations available from RDP, 16SrDNA sequence data from type strains available through LPSN downloaded, trimmed and aligned to sequences from detected sequence variants. Species names were assigned to a sequence variant when only 16S rRNA gene fragments of previously described isolates of a single species were aligned with a maximum of two mismatches with this sequence variant [6].

Protocol for virome DNA extraction

The fecal material (300 mg of fecal sample or 500 mg of fecal sample preserved in RNAlater) is resuspended in a 2 ml Eppendorf tube with 1 ml of sterile PBS buffer ((http://cshprotocols.cshlp.org/content/2006/1/pdb.rec8247) disrupted with a small spatula and vortexed until all the material that can be dissolved is in solution (~5 minutes). The diluted sample is filtered using a 10 μM filter (Sterile Syringe Filter; Hawach Scientific; Part.No.: SLPP33100S; PP 10 μm). If the filter is blocked, the filter is replaced and filtering continued. If the filter is again blocked, the not yet filtered sample (the not yet filtered sample is centrifuged at 2000 g for 5 min and the supernatant passed through a 10 μM filter previously soaked with 500μl PBS. This filtrate may be used for an additional filtering through a 0.45 μM filter (Millex-HP Syringe Filter Unit 0.45 μm, polyethersulfone, 33 mm, gamma sterilized SLHPR33RS soaked with 250 μl PBS). The filtrate of either step is supplemented with 0.1 vol of lysozyme (10 mg/mL) and incubated for 30 min at 37 °C followed by the addition of 0.2 vol of chloroform, gentle mixing and incubation for 10 min. The samples are then centrifuged at 2500 g for 5 min at room temperature for phase separation and the aqueous phase is collected. The recovered aqueous phase is supplemented with 7 U of DNaseI (Baseline-Zero DNase, Biosearch Technologies Cat.No DB071K) and 0.1 vol of 10-fold DNase buffer (50 mM $MgCl_2$, 10 mM $CaCl_2$) followed by incubation for 1 h at 37 °C and inactivation of the enzyme at 65 °C for 15 min. The resulting lysed, endonuclease treated filtrate is passed once through a 0.22 μm pore filter once (Millex-GP, 0,22 μm, Polyethersulfon, 33 mm, gamma sterilized, SLGP033RS pre-soaked with 250 μl PBS

For concentration of bacteriophage particles, the filtrate is transferred into the upper reservoir of an Amicon ultra-15 50K filter device (Merck UFC 9050) and centrifuged at 4000 g for 20 min. This time must be increased if higher volumes are to be concentrated (1 min per additional ml). Centrifugation is repeated until approximately 150 μl of volume is left. Complete dry off of the column should be avoided. Collect the volume left inside. If needed, PBS buffer is added to reach a working volume of 150 μl.

The DNA of the concentrated bacteriophage solution is extracted using the EURX kit (GeneMATRIX Tissue & Bacterial DNA purification kit Cat.No 3551). Thirty 30 µl of activation Buffer T is added onto the DNA binding spin-column and kept it at room temperature until transfer of the lysate to the spin-column. The concentrated bacteriophage solution (~150 µl) is supplemented with 375 µl of buffer Lyse T, 3 µl of RNase A and 30 µl of Proteinase K, gently vortexed and incubated at 56°C for 20 min. After gently vortexing for 7 min, 525 µl of buffer Sol T is added followed by vortexing and incubation for 10 min at 70°C. Subsequently, 525 µl of ethanol (96–100%) are added, mix thoroughly by several times inverting the tube and centrifugation for 1 min at 12000 g. The whole reaction volume is transferred to the DNA binding spin-column placed in the collection tube followed by centrifugation for 1 min at 11000 g and at maximum speed for another minute. The spin-column is removed, the flow-through discarded and the spin-column placed back into the collection tube. Subsequently, 500 µl of Wash TX1 buffer are added followed by centrifugation for 1 min at 11000 g. The flow-through is discarded and the washing step repeated by adding 500 µl of Wash TX2 buffer to the binding spin-column and centrifugation for 1 min at 11000 g. Again, the flow-through is discarded and the spin-column subject to a 1 min centrifugation at 11000 g to remove traces of Wash TX2 buffer. The spin-columns inside the collection tubes are dried in an incubator at 37 °C or above (max 60 °C) for 5 minutes. Afterwards the DNA binding spin-column is placed in a new collection tube and 50 µl of Elution buffer are added. After incubation for 2 min at room temperature the fecal virome is collected by centrifugation for 1 min at 11000 g and stored at -20°C.

Analysis of virome sequence data

The paired-end reads were analyzed with Nephele [7] a standardized set of scripts for data quality filtering, merging of paired read, assembly of contigs, and taxonomic and functional annotation. The classified fraction of the DNA sequenced was almost exclusively affiliated to bacterial species commonly found in the gut microbiome. The bacterial taxonomic composition as determined with the Nephele WGAS2 pipeline was always similar for libraries prepared from the same source sample but differing by the inclusion of an additional filtering through a 0.45 µm pore filter (10 stool samples collected at different days from 2 donors and 2 UC patients, see **Supplementary Table 4** at https://www.doi.org/10.5281/zenodo.13221182). Also, there was no difference in the percentage of sequence reads that could not be affiliated (NA) to a known bacterial genome or bacterial taxon (13.2 ± 2.1% of sequences when samples had been filtered through a 10 µm pore filter versus 11.2 ± 3.9% when samples had been filtered through an additional 0.45 µm pore filter). This fraction of non-bacterial unclassified

sequences is likely to contain a large fraction of gut virome sequences (unclassified bacteriophages) [8].

Obviously the DNA obtained still comprises a large fraction of bacterial DNA, however no additional purification attempts were included to avoid lowering total virome sequence content. Also the 0.45 µm filtering step was removed from the protocol as it does not improve the virome content. Therefore the mild 10 µm filtering step alone was the method of choice and applied to analyze the virome content in this pilot study.

The non-assembled reads were further analyzed by KAIJU [9] using the RefSeq viral database of NCBI, one of the largest repositories of viral reference sequences, where, however, gut viromes are still underrepresented. Only a minor fraction < 1% of the single reads were indicated to belong to known viral lineages and the majority of reads remained without any further classification.

Sequences were assembled by SPAdes v3.10.0 [10]. Non-classified scaffolds >10kb were extracted and used as input for Virsorter, one of the most accurate and effective pipelines to detect sequences of viral origin in metagenomic datasets. Several contigs were fulfilling the requirements of Category 1 (most confident) and thus represent near complete novel viral genome sequences of unknown taxonomy.

Mapping of sequence reads against the Cenote human virome database

As we aim to also detect variants of described viruses, we used a relaxed mapping approach [11] in Geneious Mapper (Mapper https://www.geneious.com/features/assembly-mapping/). The applied parameters were a maximum of 10% per read, minimum overlap of 25 bp, word length of 90, ignoring words repeated more than 10 times, maximum mismatches per read 15%, maximum gap size of 4 bp, minimum overlap identity of 65, index word length 14, maximum ambiguity 4, not using fine tuning, nor finding structural variants, insertions or deletions of any size. The following mapping data were extracted per sample for each of the 45033 CHVD viral reference genomes: the number of paired-end reads mapped (R), length of the reference genome in base pairs (L), the percentage of pairwise identity of the reads mapped (I), the percentage of reference genome sequence coverage (C), and the total number of reads mapped against the CHVD database (T). The relative frequency of each viral genome in a given sample was calculated as (R x L x I x C) / T.

Reproducibility of virome analysis

To study the reproducibility of virome analysis, 8 samples from donors and UC patients were selected (see **Supplementary Table 4** at https://www.doi.org/10.5281/zenodo.13221182) and subjected to the same experimental and bioinformatic procedures as outlined above. An

aliquot was subjected to an additional filtration step through a 0.45 μm pore filter. Similar virome compositions from the same sample were obtained with Bray-Curtis similarities of 82.5 ± 4.4% indicating a high reproducibility. The additional 0.45 μM pore filter step was avoided in future analyses.

Analyses of abundant phage types

CRISPR spacer-based bacterial host predictions were performed using CrisprOpenDB [12]. Lifestyle was determined with Graphage tool (https://github.com/deepomicslab/GraPhage) and topology predicted with Cenote-Taker2 [13]. Additional host prediction was performed using BLASTN vs complete non redundant reference bacterial genomes in PhageScope [14]. The host taxonomy of the hit with the lowest E-value at genus or species level (threshold of E-value < 1) was used as the predicted taxonomy. For those sequences without hits by BLASTN, DeepHost [15] was used to infer the host taxonomy on species rank using a retrained Deephost model based on 91,916 phage sequences from 413 bacterial species with a validation accuracy of 83%.

Supplementary References

1. Youngster I, Mahabamunuge J, Systrom HK, Sauk J, Khalili H, Levin J *et al.* Oral, frozen fecal microbiota transplant (FMT) capsules for recurrent *Clostridium difficile* infection. *BMC Med*. 2016;**14**:134 https://doi.org/10.1186/s12916-016-0680-9
2. Ott SJ, Waetzig GH, Rehman A, Moltzau-Anderson J, Bharti R, Grasis JA *et al.* Efficacy of sterile fecal filtrate transfer for treating patients with *Clostridium difficile* infection. *Gastroenterology*. 2017;**152**:799-811 e7 https://doi.org/10.1053/j.gastro.2016.11.010
3. Cole JR, Wang Q, Fish JA, Chai B, McGarrell DM, Sun Y *et al.* Ribosomal database project: Data and tools for high throughput rRNA analysis. *Nucleic Acids Res*. 2014;**42**:D633-42 https://doi.org/10.1093/nar/gkt1244
4. Quast C, Pruesse E, Yilmaz P, Gerken J, Schweer T, Yarza P *et al.* The SILVA ribosomal RNA gene database project: Improved data processing and web-based tools. *Nucleic Acids Res*. 2013;**41**:D590-6 https://doi.org/10.1093/nar/gks1219
5. Parte AC, Sarda Carbasse J, Meier-Kolthoff JP, Reimer LC, Goker M. List of prokaryotic names with standing in nomenclature (LPSN) moves to the dsmz. *Int J Syst Evol Microbiol*. 2020;**70**:5607-12 https://doi.org/10.1099/ijsem.0.004332
6. Schulz C, Schutte K, Koch N, Vilchez-Vargas R, Wos-Oxley ML, Oxley APA *et al.* The active bacterial assemblages of the upper gi tract in individuals with and without

Helicobacter infection. *Gut*. 2018;**67**:216-25 https://doi.org/10.1136/gutjnl-2016-312904

7. Weber N, Liou D, Dommer J, MacMenamin P, Quinones M, Misner I *et al.* Nephele: A cloud platform for simplified, standardized and reproducible microbiome data analysis. *Bioinformatics*. 2018;**34**:1411-13 https://doi.org/10.1093/bioinformatics/btx617
8. Gregory AC, Zablocki O, Zayed AA, Howell A, Bolduc B, Sullivan MB. The gut virome database reveals age-dependent patterns of virome diversity in the human gut. *Cell Host Microbe*. 2020;**28**:724-40 e8 https://doi.org/10.1016/j.chom.2020.08.003
9. Menzel P, Ng KL, Krogh A. Fast and sensitive taxonomic classification for metagenomics with Kaiju. *Nat Commun*. 2016;**7**:11257 https://doi.org/10.1038/ncomms11257
10. Bankevich A, Nurk S, Antipov D, Gurevich AA, Dvorkin M, Kulikov AS *et al.* Spades: A new genome assembly algorithm and its applications to single-cell sequencing. *J Comput Biol*. 2012;**19**:455-77 https://doi.org/10.1089/cmb.2012.0021
11. Milian-Garcia Y, Hempel CA, Janke LAA, Young RG, Furukawa-Stoffer T, Ambagala A *et al.* Mitochondrial genome sequencing, mapping, and assembly benchmarking for *Culicoides* species (diptera: Ceratopogonidae). *BMC genomics*. 2022;**23**:584 https://doi.org/10.1186/s12864-022-08743-x
12. Dion MB, Plante PL, Zufferey E, Shah SA, Corbeil J, Moineau S. Streamlining CRISPR spacer-based bacterial host predictions to decipher the viral dark matter. *Nucleic Acids Res*. 2021;**49**:3127-38 https://doi.org/10.1093/nar/gkab133
13. Tisza MJ, Belford AK, Dominguez-Huerta G, Bolduc B, Buck CB. Cenote-taker 2 democratizes virus discovery and sequence annotation. *Virus Evol*. 2021;**7**:veaa100 https://doi.org/10.1093/ve/veaa100
14. Wang RH, Yang S, Liu Z, Zhang Y, Wang X, Xu Z *et al.* Phagescope: A well-annotated bacteriophage database with automatic analyses and visualizations. *Nucleic Acids Res*. 2024;**52**:D756-D61 https://doi.org/10.1093/nar/gkad979
15. Ruohan W, Xianglilan Z, Jianping W, Shuai Cheng LI. Deephost: Phage host prediction with convolutional neural network. *Brief Bioinform*. 2022;**23** https://doi.org/10.1093/bib/bbab385

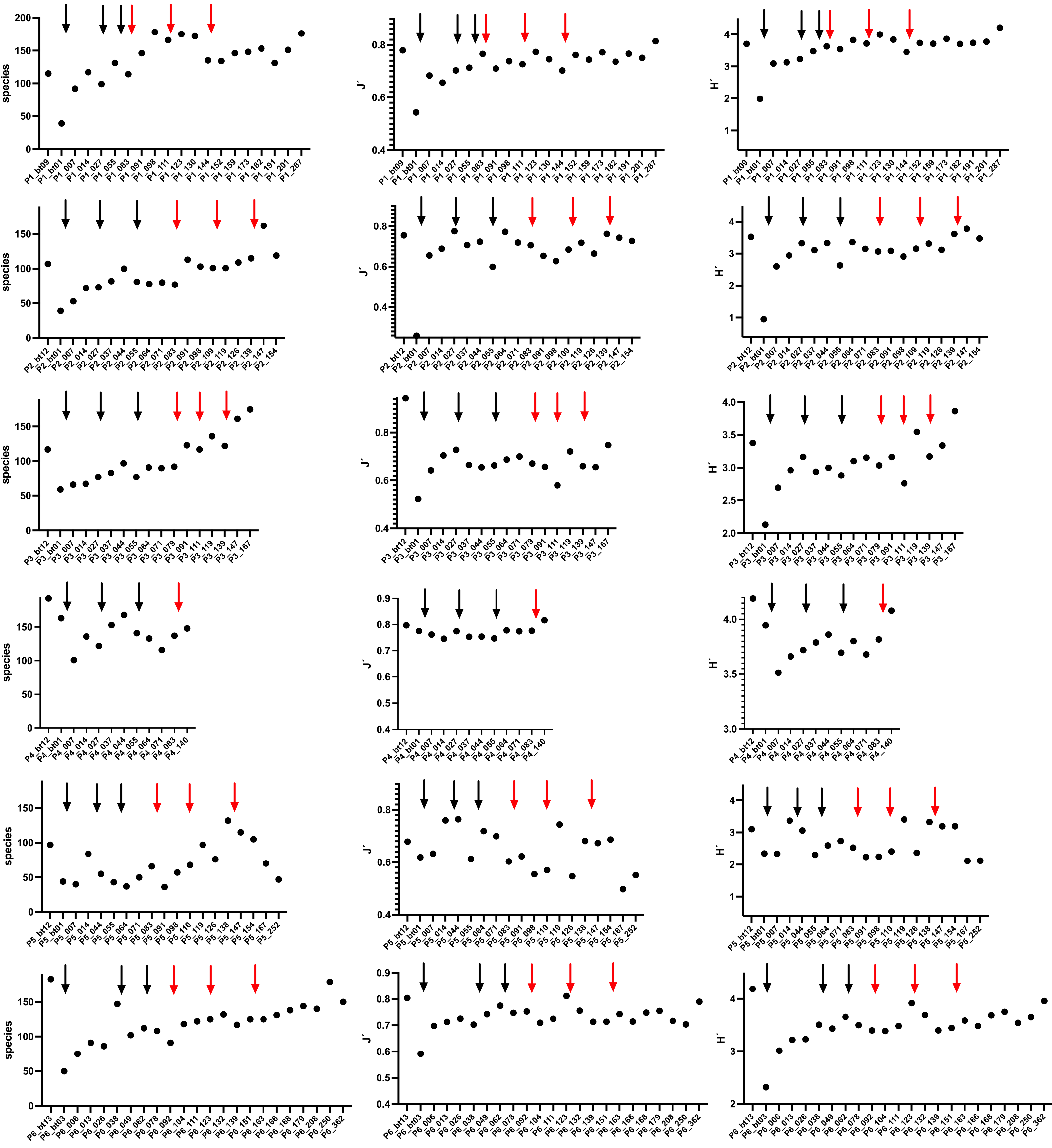

Supplementary Figure S1. Bacterial community diversity in patients P1 - P6 during treatment time. Diversity is indicated by total species number (taxa), Pielou's evenness (J´) and Shannon diversity (H´), respectively, and was analyzed using species level taxon relative abundance data as input. The time of FMFT is indicated by a black arrow, the time of FMT by a red arrow. The time in days before treatment (bt) as well as after treatment start is given.

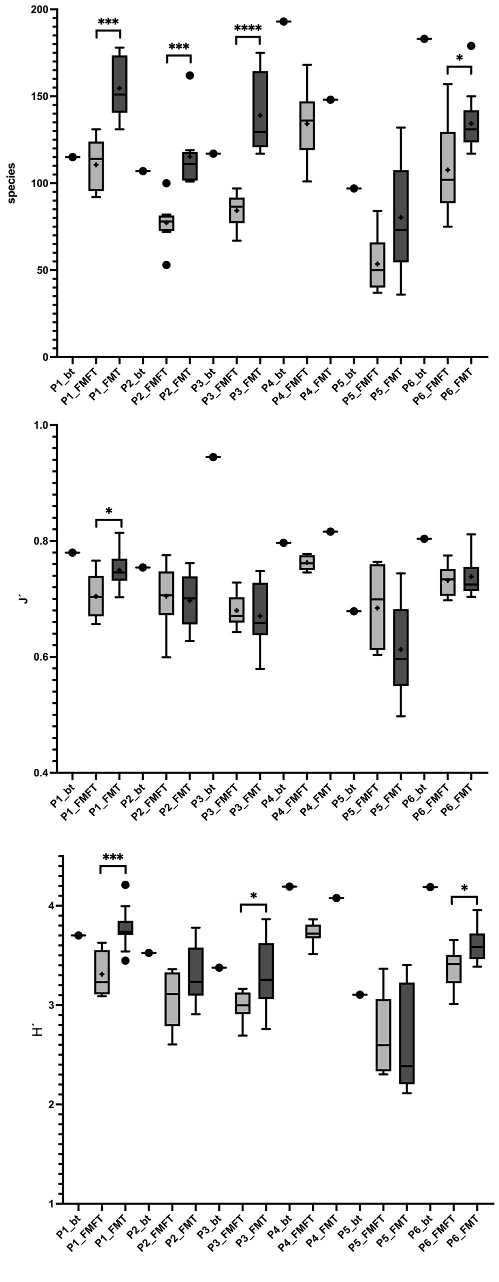

Supplementary Figure S2. Bacterial community diversity in patients P1 - P6 before (bt), during FMFT (f) and during FMT (m). Diversity is indicated by total species number (taxa), Shannon diversity (H´) and Pielou's evenness (J´), respectively, and was analyzed using species level taxon relative abundance data as input. Statistically significant differences in diversity of communities during FMFT and FMT are indicated as *$p < 0.05$, **$p < 0.01$, ***$p < 0.001$, or ****$p < 0.0001$. The mean is indicated by + and the median by a black line. The box represents the interquartile range. The whiskers extend to the upper adjacent value (largest value = 75th percentile +1.5 IQR) and the lower adjacent value (lowest value = 25th percentile -1.5 IQR) and dots represent outliers. In case of community structure before treatment, only one or two timepoints were available such that no statistical evaluation was performed.

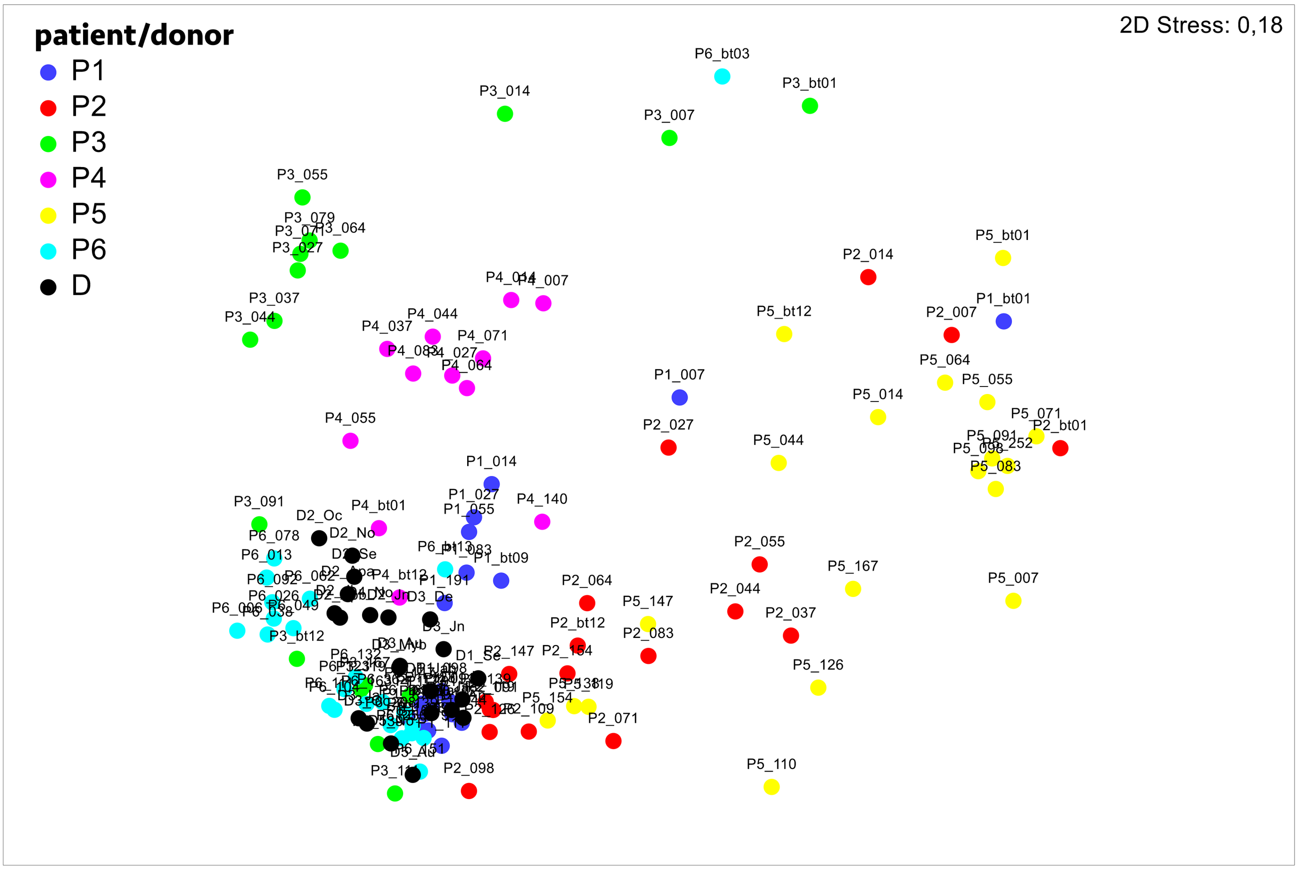

Supplementary Figure S3. Differences in global bacterial community structures. The global bacterial community structures in patients P1 – P6 and Donor D1 – D5 were assessed by non-metric multidimensional scaling (nMDS) and are based on standardized species abundance data. Similarities were calculated using the Bray–Curtis similarity algorithm. The treatment time (in days) is indicated relative to the start of treatment (first FMFT), with bt indicating days before treatment.

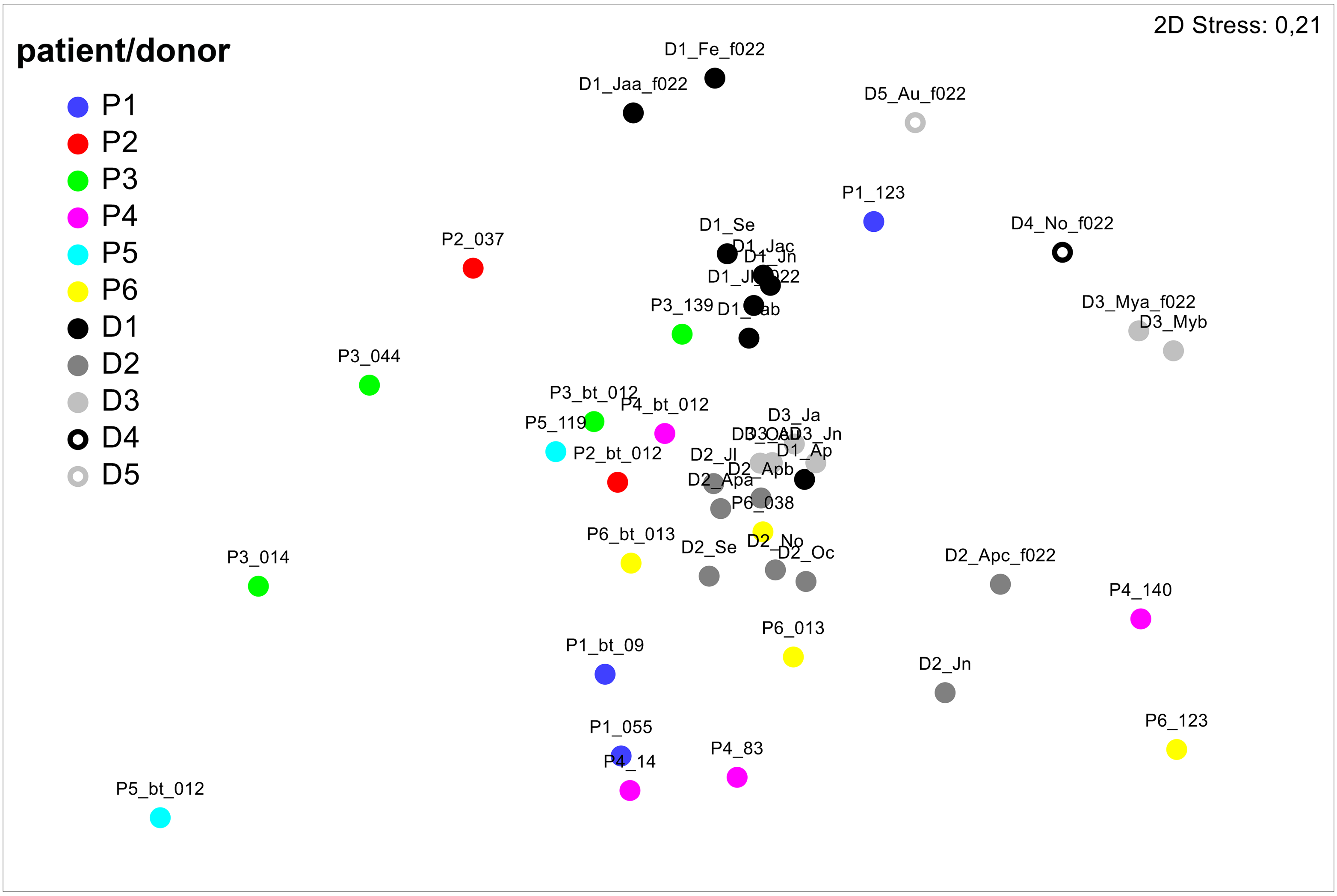

Supplementary Figure S4. Differences in global virome structures of patients and donors. The global virome structures in patients P1 – P6 and Donor D1 – D5 were assessed by non-metric multidimensional scaling (nMDS) and are based on relative abundance data. Similarities were calculated using the Bray–Curtis similarity algorithm. The treatment time (in days) of patients is indicated relative to the start of treatment (first FMFT), with bt indicating days before treatment. The month of donor sampling in 2019 is indicated by a 3-letter code. Filtrates were prepared either via a standard protocol or a protocol including an additional initial filtering through a 0.22 M pore filter (f022) designed for application to patients.
